# Virulence and the presence of aminoglycoside resistance genes of *Staphylococcus haemolyticus* strains isolated from clinical specimens

**DOI:** 10.1007/s10482-015-0378-6

**Published:** 2015-01-15

**Authors:** Sylwia Krzymińska, Ewa Szczuka, Kinga Dudzińska, Adam Kaznowski

**Affiliations:** Department of Microbiology, Faculty of Biology, A.Mickiewicz University, ul. Umultowska 89, 61-614 Poznan, Poland

**Keywords:** *Staphylococcus haemolyticus*, Antibiotic resistance, Adhesion, Invasion, Cytotoxicity, Apoptosis, Epithelial cells

## Abstract

We examined thirty methicillin-resistant *Staphylococcus haemolyticus* isolates cultured from clinical specimens for antibiotic resistance, various important interactions of the bacteria with epithelial cells and putative virulence determinants. All strains were resistant to oxacillin and carried the *mecA* gene. Aminocyclitol-3′-phosphotransferase (*aph*(3′)*-IIIa*) gene encoding nucleotidyltransferases was detected in 43 %, aminocyclitol-6′-acetyltransferase-aminocyclitol-2″-phosphotransferase (*aac*(6′)/*aph*(2″)) gene encoding bifunctional acetyltransferases/phosphotransferases in 33 %, aminocyclitol-4′-adenylyltransferase (*ant*(4′)-*Ia*) gene encoding phosphotransferases in 20 %. The coexistence of resistance to methicillin and aminoglycosides was investigated in multi-resistant strains. Coexisting (*aac*(6′)/*aph*(2″)) and (*aph*(3′)-*IIIa*) genes were detected in 33 % of isolates, whereas 63 % of isolates had at least one of these genes. All strains revealed adherence ability and most of them (63 %) were invasive to epithelial cells. Electron microscopy revealed that the bacteria were found in vacuoles inside the cells. We observed that the contact of the bacteria with host epithelial cells is a prerequisite to their cytotoxicity at 5 h-incubation. Culture supernatant of the strains induced a low effect of cytotoxicity at the same time of incubation. Cell-free supernatant of all isolates expressed cytotoxic activity which caused destruction of HEp-2 cells at 24 h. None of the strains was cytotonic towards CHO cells. Among thirty strains, 27 % revealed lipolytic activity, 43 % produced lecithinase and 20 % were positive for proteinase activity. Analyses of cellular morphology and DNA fragmentation exhibited typical characteristic features of those undergoing apoptosis. The Pearson linear test revealed positive correlations between the apoptotic index at 24 h and percentage of cytotoxicity. Our results provided new insights into the mechanisms contributing to the development of *S*. *haemolyticus*-associated infections. The bacteria adhered and invaded to non-professional phagocytes. The invasion of epithelial cells by *S. haemolyticus* could be similar to phagocytosis that requires polymerization of the actin cytoskeleton. The process is inhibited by cytochalasin D. Moreover, they survived within the cells by residing in membrane bound compartments and induced apoptotic cell death.

## Introduction

Coagulase-negative staphylococci (CNS) are a group of bacteria that are increasingly implicated as a cause of hospital-acquired opportunistic and health-care related infections worldwide. *Staphylococcus haemolyticus* strains are the second of the most frequently encountered CNS that have been recognized as an emerging and important human pathogen causing serious infections such as: endocarditis, urinary tract infections, septicemia, peritonitis, wound, bone and joint infections. Patients with the illnesses are usually immunocompromised, with indwelling or implanted foreign bodies (Piette and Verschraegen 2009).

An important feature of *S*. *haemolyticus* strains is resistance to β-lactams and other antibiotics agents (Götz 2006). The treatment of methicillin-resistant staphylococci is generally based on glycopeptides, especially vancomycin. When combination therapy is required, the aminoglycosides are used because of their killing potential and the postantibiotic effect. Significantly, the aminoglycosides have an ability to produce synergistic bactericidal activity in combination with antimicrobial agents inhibiting cell wall biosynthesis, including vancomycin (Vakulenko and Mobashery 2003). In staphylococci resistant to aminoglycosides, it is commonly due to drug inactivation by cellular aminoglycosides-modifying enzymes (You et al. 2000). The bifunctional enzyme AAC(6′)/APH(2″), encoded by *aac*(6′)/*aph*(2″) mediates resistance to gentamicin, neomycin, kanamycin, tobramycin and amikacin. The APH(3′)-III enzyme encoded by *aph*(3′)-IIIa, essentially modified kanamycin and amikacin, whereas the ANT(4′)-I enzyme encoded by *ant*(4′)-*Ia* inactivates tobramycin, kanamycin, neomycin and amikacin (Schmitz et al. 1999).

Although *S. haemolyticus* strains are among the most common CNS species causing hospital-acquired opportunistic infections, little is known about their virulence-associated properties. Takeuchi et al. (2005) analysed the whole genome sequence of human pathogenic strain and reported 57 open reading frames (orfs) associated with virulence. They identified numerous genes encoded putative enzymes and toxins. At least three orfs showed homology to staphylococcal α-hemolysins, *Bacillus cereus* and streptococcal hemolysins. The *S*. *haemolyticus* genome carried genes which encode enzymes for synthesis of the poly-gamma-glutamate capsule that protects against cationic microbiological peptides (Takueschi et al. 2005). The exact role of these cellular and extracellular products in the pathogenesis of *S*. *haemolyticus* strains is still unclear.

The putative mechanism of *S. haemolyticus* pathogenesis remains poorly documented. Therefore in the study, we analysed possible virulence factors of the methicillin-resistant *S. haemolyticus* clinical strains and assessed the interaction of the bacteria with epithelium. We investigated adherence, invasion, cytotoxic and apoptotic activity of the isolates to HEp-2 cells. Moreover, we determined susceptibility of the strains to antibiotics with a focus on aminoglycoside resistance.

## Materials and methods

### Bacterial strains and growth conditions

We evaluated 30 methicillin-resistant *S*. *haemolyticus* isolates that were previously identified to the species level and clonal analysed by REP-PCR typing (Krzymińska et al. 2012a). The strains originated from clinical samples: blood (15), wounds (4), respiratory secretions (4), skin (2), urine (2) and medical devices (Table 1). They were collected from hospitalized patients over a 2-year period, as described earlier. These strains and *Escherichia coli* as the negative control were grown overnight in tryptic soy broth (TSB, Difco) 37 °C. The cultures were centrifuged at 2,000×*g* for 20 min. Supernatants were sterilized through 0.22 μm-pore size membrane filters Millex-GV (Millipore) and heated (56 °C for 20 min) to destroy the activity of heat-labile toxins. For the analysis of cell-contact cytotoxicity, the pellet of bacterial cells were resuspended in PBS and adjusted to the optical density OD of 0.4, corresponding to 0.8–1.5 × 10^4^ colony- forming units (CFU/1 ml).

**Table 1:** Source of origin and aminoglycoside resistance genes of *Staphylococcus haemolyticus* strains

Strain no.	Source of origin	Aminoglycoside resistance genes		
		*aph*(3′)-*III*	*ant*(4′)-*Ia*	*aac*(6′)*/aph*(2″)
MPU Sh1	Blood	+	−	+
MPU Sh2	Secretion	+	−	+
MPU Sh3	Urine	−	−	−
MPU Sh4	Wound	+	−	−
MPU Sh5	Urine	−	−	−
MPU Sh6	Blood	+	−	+
MPU Sh7	Blood	−	−	−
MPU Sh8	Secretion	−	+	−
MPU Sh9	Wound	+	−	+
MPU Sh10	Medical devices	+	−	−
MPU Sh11	Blood	+	−	+
MPU Sh12	Medical devices	−	−	−
MPU Sh13	Blood	−	+	−
MPU Sh14	Skin	−	−	−
MPU Sh15	Wound	−	−	−
MPU Sh16	Blood	−	+	−
MPU Sh17	Blood	−	−	−
MPU Sh18	Secretion	+	−	+
MPU Sh19	Blood	+	−	−
MPU Sh20	Blood	−	−	−
MPU Sh21	Medical devices	+	−	+
MPU Sh22	Blood	−	+	−
MPU Sh23	Blood	−	−	−
MPU Sh24	Blood	+	−	+
MPU Sh25	Blood	+	−	+
MPU Sh26	Blood	−	+	−
MPU Sh27	Skin	−	−	−
MPU Sh28	Wound	+	−	+
MPU Sh29	Secretion	−	−	−
MPU Sh30	Blood	−	+	−

### Antibiotic susceptibility testing

Susceptibility to the following antibiotical agents: ciprofloxacin, clindamycin, erythromycin, gentamycin, tobramycin, oxacillin, rifampicin, teicoplanin, tetracycline, trimethoprim/sulfamethoxazole, vancomycin, levofloxacin, norfloxacin and linezolid was performed using the Vitek 2 system (bioMérieux, France).

### Identification of aminoglycoside resistance genes

Isolation of DNA was performed by using the Genomic DNA Plus kit (A&A Biotechnology, Poland). A multiplex PCR was applied for simultaneous amplification of *aac*(6′)/*aph*(2″), *aph*(3′)-*IIIa*, *ant*(4′)-*Ia* gene. The PCR amplification of the *mecA* genes was performed as described previously by Ardic et al. (2006). The amplification products were electrophoresed in 1.5 % agarose gel, stained with EB, visualized on a UV light transilluminator, and documented with V.99 Bio-Print system (Vilber Lourmat, Torcy, France).

### Epithelial cell line

Chinese hamster ovary cells (CHO) and human epidermoid carcinoma cells from the larynx (HEp-2) were cultured in Eagle minimal essential medium (GM, Sigma) with 5 % fetal calf serum (FCS, Sigma) containing 2 mM glutamine, penicillin (50 Iu/ml), streptomycin (100 μg/ml) and 1 mg/ml of nystatin (Krzymińska et al. 2010).

### Infection of HEp-2 cells

The epithelial cells were seeded in culture 96-well plates and were grown in GM, until 80 % of confluency was obtained. Next, the medium was replaced with Eagle medium without FCS and antibiotics for 24 h. During infection, the monolayer was incubated with bacterial suspension at a multiplicity of infection (MOI) of 10 bacteria per HEp-2 cell (the cells in number 1 × 10^5^ incubated with approximately of 1 × 10^6^ of bacteria) for 2 h at 37 °C to allow for adhesion and invasion of bacteria to epithelial cells.

### Adhesion and invasion assay

Adhesion and invasion of *S. haemolyticus* to human epithelial cells were quantified using gentamicin, lysostaphin protection assay with modifications (Agerer et al. 2005; Rocha-de-Souza et al. 2008). Monolayer of HEp-2 cells in two sets of triplicate wells was incubated with the bacteria for 2 h. During the incubation the relative growth rate was approximately equal for each strain. To enumerate total associated (adhered and intracellular) bacteria, one set of triplicate wells of infected cells was washed three times with PBS and lysed in 100 μl 0.1 % Triton X-100. For invasion assay, a second set of triplicate wells with infected cells was washed with PBS and incubated with Eagle medium containing 300 μg/ml of gentamicin and 20 μg/ml lysostaphin for 2 h. After washing three times with PBS, the infected cells were lysed with 0.1 % Triton X-100 in PBS. The lysates were serially diluted and plated on TSA and enumerated by colony-forming units (CFU) counting. The number of attached bacteria was determined by subtracting the intracellular bacteria following invasion from the total number of adhered and invaded once. Bacterial adhesion and invasion ability were presented as respectively the adhesion (AdI) and invasion (InI) indexes. The adhesion index was expressed as the mean total number of CFU associated bacteria per well (1 × 10^5^ HEp-2 cells). The invasion index (InI) presented as the number of internalised bacteria per well after gentamicin/lysostaphin treatment. To determine whether the cytoskeleton of epithelial cells is involved in bacterial uptake, the cells were treated with cytochalasin D (to inhibit actin polymerization). In parallel experiments, cytochalasin D (1 μg/ml, Sigma), was added to the cells 30 min prior to infection. Addition of cytochalasin D during the experiment had no effect on the bacterial and epithelial cell viability. The monolayer was infected separately with an invasive strain of *Yersinia enterocolitica* O:3/4 (pYV^+^) and non-pathogenic *E. coli* K-12 C600.

### Cell-contact and extracellular toxic activities

Cytotoxic activity to epithelial cells was measured in a bacterial cell suspension and extracellular culture filtrates. In order to determine cell-contact cytotoxicity, epithelial cells were grown in six-well plates containing 1 ml of GM per well. The bacterial cell suspension in GM at a MOI of 10:1 was added to each well and separately to transwell cell culture chamber inserts (Nunc). The membrane at the bottom of the insert precludes direct interaction between bacteria and epithelial cells. However, it allows for the exchange of extracellular products. The monolayer of HEp-2 cells was incubated with the bacterial cell suspension for 5 h and cell-free culture supernatant for 5 and 24 h at 37 °C. The cytotoxic activity was assessed in Neutral Red (NR) retention assay (Krzymińska et al. 2010). Cytotonic activity of cell associated and extracellular factors was observed on CHO cells.

### Assessment of apoptosis

Apoptosis of infected epithelial cells was assessed by three different methods: trypan blue exclusion assay, double staining with acridine orange (AO, 100 μg/ml)/ethidium bromide (EB, 100 μg/ml) and an analysis of nuclear DNA fragmentation. Viability of the cells was tested in the trypan blue exclusion assay (Krzymińska et al. 2010). Morphological evidence of apoptosis and necrosis was obtained by means of AO and EB staining (Krzymińska et al. 2012a, b). The cells were examinated with a Zeiss Axiovert 405 M inverted microscope equipped with epifluorescence. Confocal images of green fluorescence were collected using a 488 excitation and 514–540 band-pass filter. Images of red fluorescence were collected using a 568 nm excitation and 590 long pass filter. Images were acquired using a Zeiss AxioCam HRc camera operating under AxioVision 3,1 software, and next processed using Adobe Photoshop CS5. The cells were recorded in three different groups: viable, apoptotic and necrotic cells. Viable (green), apoptotic (green with red nuclei) and necrotic cells (red) were quantified by counting a minimum of 100 cells in three independent experiments. The results were presented as the percentage of apoptotic cells [Apoptotic Index (AI)] and the percentage of necrotic cells (Necrotic Index). A biochemical hallmark of apoptosis is fragmentation of DNA that was extracted from the infected cells (Krzymińska et al. 2012b).

### Transmission electron microscopy (TEM)

Electron microscopy was used to evaluate morphological changes of HEp-2 cells incubated with *S. haemolyticus* strains. The cells, after 3 h-infection, were washed three times with PBS and harvested by trypsinization. Next, they were centrifuged at 1,500×*g* for 15 min, fixed with 2.5 % glutaraldehyde in 0.1 M phosphate buffer for 1 h, washed with phosphate buffer, postfixed with 1 % OsO_4_, gradually dehydrated in series of acetone, and embedded in Epon (Choi et al. 2005). The samples were sliced into 70-nm sections, stained with uranyl acetate and examined with a transmission electron microscope (JEM 1200 EXII) TEM at accelerating voltage of 80 kV.

### Determination of exoenzymes activity

We analysed the activity of extracellular enzymes that may act as putative virulence factors: lipase, lecithinase (phospholipase) and serine protease. Lipase activity was detected on TSB agar (TSA) enriched with 0.01 % CaCl_2_ × 2H_2_O and 1 % Tween 80 (Difco). Lipolytic activity was indicated by the appearance of a precipitation zone resulting from the deposition of crystals formed with calcium salt and fatty acids generated by the enzyme (Kumar et al. 2012). Lecithinase activity was examined on BHI agar supplemented with 10 % (vol/vol) egg-yolk (Karasawa et al. 2003). Inoculated plates were incubated for 48 h at 35 °C and 24 h at room temperature. Lecithinase activity was characterized by a transparent zone around colonies. Production of serine protease was determined on TSA supplemented with 5 % (w/v) casein (Lucas and Manna 2013). The activity was observed as a transparent zone surrounding the bacteria colonies.

### Statistical analysis

The data were presented as mean ± standard deviation from two independent experiments performed in duplicate. A one-way analysis of variance ANOVA with Tukey’s post hoc test at the significance level P < 0.05 was performed. The linear regression analysis was used to examine pairwise correlation between the AI, cytotoxicity, the Pearson correlation coefficient was determined. P values of <0.05 were considered statistically significant. The statistical analysis was performed using Statistica PL software (StatSoft Poland Inc., USA).

## Results

### Antibiotic resistance of *S. haemolyticus* strains

All *S. haemolyticus* strains were resistant to oxacillin. The *mecA* gene was present in all the strains. Resistance to vancomycin, teicoplanin and linezolid was not detected. Analysis of the antibiotic resistance of the strains showed that all of them were multiresistant i.e., expressing resistance to more than three different classes of antibiotics, in addition to β-lactams. Erythromycin resistance was found for 28 (93 %) strains. We observed resistance to gentamycin for 40 % of strains, tobramycin (33 %), trimethoprim/sulfamethoxazole (56 %), clindamycin (50 %) and tetracycline (36 %). Only two strains (7 %) exhibited resistance to rifampicin. Resistance to ciprofloxacin, norfloxacin and levofloxacin was revealed respectively by 53, 36 and 30 % of the strains.

### The presence of aminoglycoside resistance genes

The PCR analysis revealed that aminoglycoside resistance was due to the existence of gene *aph*(3′)-*IIIa* for 43 % of the strains, *aac*(6′)/*aph*(2″) (33 %), and *ant*(4′)-*Ia* (20 %). Ten isolates (33 %) contained two AME genes, *aac*(6′)/*aph*(2″) and *aph*(3′)-*IIIa,* simultaneously (Table 1). At least one AME was encountered in 19 (63 %) of *S. haemolyticus* strains, whereas 11 strains did not harbour any of the AME. It is noteworthy that among the 12 gentamicin-resistant strains, two strains did not harbour *aac*(6′)/*aph*(2″). All gentamicin-susceptible *S. haemolyticus* isolates were negative by PCR for the bifunctional gene.

In Table 2 we present summarized results of assessment of *S. haemolyticus* apoptotic, cytotoxic activities, adhesion, invasion to HEp-2 cells, and enzyme activity that might contribute to the virulence.

**Table 2:** Apoptotic index, cytotoxic, adhesion, invasion activities and production of lipase, lecithinase and proteinase by *S. haemolyticus* strains

Strain no	Apoptotic index^a^ (%)		Cytotoxicity (%)		Adhesion index^d^(×106)	Invasion index^e^ (%)	Exoenzyme		
	24 h	48 h	Cell-contact^b^	Extracellular^c^			L	Le	P
MPU Sh4	59.8	89.8	66.7	72.8	6.9	12.6	+	+	+
MPU Sh3	51.3	79.9	60.6	70.9	2.3	0.8	−	−	−
MPU Sh9	48.7	76.9	63.6	81.5	13.5	16.9	+	−	+
MPU Sh15	48.1	62.9	56.0	61.2	2.1	0.2	−	+	−
MPU Sh27	47.2	69.1	54.9	39.6	8.6	0.6	−	+	−
MPU Sh11	46.2	63.9	60.6	69.1	8.4	6.4	+	−	+
MPU Sh13	43.1	58.4	53.0	62.1	15.3	0.8	+	−	+
MPU Sh8	42.9	71.1	57.6	81.5	2.3	0	+	+	−
MPU Sh20	42.8	76.2	31.8	33.5	13.1	9.7	+	+	−
MPU Sh29	42.1	62.8	51.2	71.1	0.7	0	−	−	−
MPU Sh10	41.9	73.4	65.1	69.1	7.3	0.03	−	+	−
MPU Sh17	41.8	61.6	31.8	68.9	9.1	0	−	+	−
MPU Sh12	41.7	71.3	57.6	79.6	1.3	0	−	+	−
MPU Sh5	41.6	79.5	57.6	77.7	0.8	10.4	+	−	+
MPU Sh25	41.2	69.8	22.7	73.2	9.6	0.01	−	+	−
MPU Sh6	39.6	42.7	9.6	75.7	9.8	3.1	−	+	−
MPU Sh19	38.7	69.3	13.6	14.8	14.6	0.01	−	+	−
MPU Sh1	38.5	41.3	72.7	77.7	12.3	3.2	−	+	−
MPU Sh7	36.8	39.7	37.9	38.6	10.2	0	−	−	−
MPU Sh30	31.6	37.9	45.4	11.2	13.7	0	−	−	−
MPU Sh16	31.2	34.9	43.9	35.0	8.9	1.4	−	−	−
MPU Sh2	19.8	65.2	62.1	67.3	0.6	17.9	+	−	+
MPU Sh22	14.5	28.9	36.4	24.7	1.1	0	−	−	−
MPU Sh24	9.1	21.3	37.9	13.0	11.8	0.07	−	+	−
MPU Sh14	6.7	21.3	45.5	25.7	13.6	0	−	−	−
MPU Sh28	4.2	6.9	37.9	16.8	0.4	0	−	−	−
MPU Sh21	3.1	4.8	19.7	26.7	8.3	0.01	−	−	−
MPU Sh18	3.1	9.3	25.7	13.8	0.2	0.04	−	−	−
MPU Sh23	1.9	3.9	25.8	42.8	0.9	0	−	−	−
MPU Sh26	1.2	2.2	34.8	21.2	8.4	0	−	−	−

The data are the means from two separate experiments in triplicate

*L* lipase, *Le* lecithinase (phospholipase), *P* proteinase

^a^Percentage of apoptotic cells

^b,c^Percentage of cytotoxicity was determined by NR assay

^d^Mean number of associated (CFU) bacteria/1 × 10^5^ HEp-2 cells

^e^Percentage of the number of internalized bacteria compared to the number of those adhered

### *S. haemolyticus* adherence to epithelial cells

All of the *S. haemolyticus* strains adhered to human epithelial cells with the range from 2 × 10^5^ to 1.53 × 10^7^ CFU per 1 × 10^5^ cells. The highest adhesion index, ranging from 1.31 to 1.53 × 10^7^ was revealed for 6 (20 %) strains. The lowest adhesion ability, from 0.2 × 10^6^ to 1.1 × 10^6^ CFU was observed for 7 (23.3 %) isolates. The negative control of nonpathogenic *E. coli* K12C600 showed the index of 1.2 × 10^2^ CFU, whereas that of *Y. enterocolitica* O:8/1B, positive control elevated to 25.8 × 10^5^ CFU.

### *S. haemolyticus* invasion of epithelial cells

Nineteen strains (63.3 %) exhibited invasion of epithelial cells. The percentage of associated bacteria that were internalized ranged from 0.01 to 17.9 %. Three (10 %) strains showed the highest invasion activity with the invasion index from 17.9 to 12.6 %. Six (20 %) isolates exhibited the lowest invasive ability (0.01–0.07 %), higher than that of the nonpathogenic negative control. Eleven (37 %) strains did not reveal invasive ability. The percentage of invading bacteria ranged from 0.1 to 10.4 %, which was higher than the negative control, was observed for 10 (64 %) isolates. The invasion index for *Y. enterocolitica* O:8/1B reached 57.3 %, whereas for *E. coli* K12C600 strains that invaded HEp-2 cells, it was 0.003 % of total adhered cells. Treatment of epithelial cells with cytochalasin D resulted in a reduction of the bacteria uptake in the range from 84 % (±1.7 %) to 91 % (±1.9 %) after 24 h. These data suggested that actin polymerization plays a major role in bacterial internalization. Treatment of epithelial cells with cytochalasin D had no effect on *S*. *haemolyticus* adhesion to epithelial cells.

### Cell-contact cytotoxicity of *S. haemolyticus* strains

To determine whether cell contact is required for *S*. *haemolyticus*-induced cytotoxicity, infected epithelial cells were observed using inverted phase-contrast microscopy. After 5-h incubation, the cells were detached from the plate surface (results not shown). Morphological changes were confirmed by the NR test (Table 2). The cell contact-cytotoxicity was ranging from 9.6 to 66.7 %. The highest activity in the range from 53 to 66.7 % of destructed cells was revealed by 10 (30 %) strains. The lowest cytotoxicity below 10 % was observed for 1 (3.3 %) strain. Low percentage of cytotoxicity from 0.4 to 1.1 could be noticed when *S*. *haemolyticus* cells were not allowed to contact with epithelial cells in the culture inserts. The results suggested that extracellular factors may contribute to the cytotoxicity. Bacterial culture supernatants of 19 (63.3 %) strains showed a low effect (7.3 ± 2.1 %) on viability of HEp-2 cells at 5-h incubation. No cell lysis was observed at 5-h incubation with culture medium (GM) and non-pathogenic *E. coli* K-12 C600 strain.

### Extracellular cytotonic and cytotoxic activity

None of *S*. *haemolyticus* isolates revealed cytotonic activity to CHO cell line, whereas all strains revealed activity of extracellular factors which destroy HEp-2 cells. The percent of cytotoxicity ranged from 13.8 to 81.5 % at 24 h. The highest activity in the range from 72.8 to 81.5 % was observed for 8 (26.7 %) isolates. Seven (23.3 %) strains exhibited the lowest activity in the range from 11.2 to 25.7 %. The negative control *E. coli* K-12 C600 strain was not cytotoxic to epithelial cells. Preheating (56º C for 20 min) of cell-free supernatants of 23 (77 %) strains caused a reduction of cytotoxic activity to HEp-2 cells in the range from 7.1 % (±1.7 %) to 19.6 % (±2.9 %) after 24 h.

### Apoptosis of epithelial cells induced by *S*. *haemolyticus* strains

**Fig. 1:**
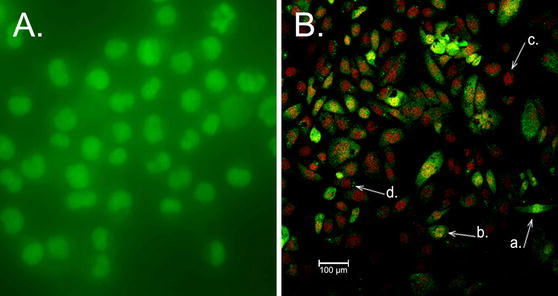
Apoptosis of HEp-2 cells due to *S. haemolyticus* infection. The cells were stained with AO/EB and observed under a laser confocal microscope. **a**—control, noninfected cells **b**—epithelial cells infected with *S. haemolyticus* MPU Sh4 at 48 h after infection; *a*—live cells, *b*—apoptotic cells, *c*—necrotic cells, *d*—intracellular bacteria

Infected epithelial cells with *S*. *haemolyticus* isolates revealed signs of apoptosis in the EB/AO staining. AO penetrates into viable cells and stain them green (Fig. 1a). EB is taken up by the cells when cytoplasmic membrane integrity is lost and colour the nuclei red (Fig. 1b). Viable (green), apoptotic (green with red nuclei) and necrotic cells (red) were quantified by counting a minimum of 100 cells in three independent experiments. Immunofluorescence photography also showed bacteria adhered and invaded to epithelial cells.

**Fig. 2:**
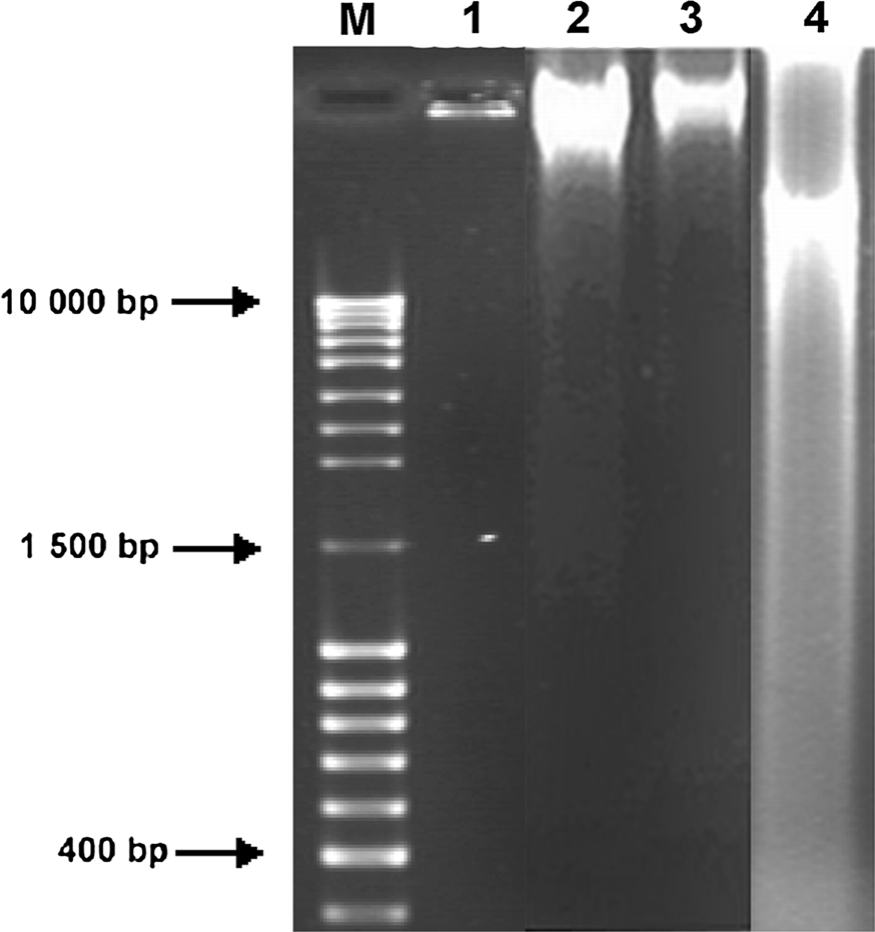
Intranucleosomal degradation of DNA from HEp-2 cells infected with different strains. DNA isolated from cells infected with: *lane 1*—*E. coli* K12C600 (negative control); *lane 2*—*S. haemolyticus* MPU Sh7 at 24 h after infection; *lane 3*—*S. haemolyticus* MPU Sh7 at 48 h after infection; *lane 4*—*S. haemolyticus* MPU Sh4 at 24 h after infection; *M*—molecular size marker

All *S*. *haemolyticus* strains induced epithelial cell death through apoptosis. The AI ranged from 1.2 to 59.8 % at 24 h after infection. The highest AI (46.2–59.8 %) at 24 h was observed in cells incubated with 6 (20 %) strains. The lowest AI, between 1.2 and 9.1 %, was expressed by 7 (23.3 %) isolates. The percentage of apoptotic cells increased in the range from 2.2 to 89.8 % at 48 h post infection. The highest AI ranging from 76.2 to 89.8 % was observed in HEp-2 cells infected with 5 (16.7 %) of the *S*. *haemolyticus* strains. The lowest AI ranging from 2.2 to 21.3 % was revealed by cells infected with 7 (23.3 %) of the strains. The mean AI of the negative control was 4.1 **±** 1 %, whereas for the positive control it reached 94.6 ± 5.1 %. Some *S*. *haemolyticus* strains also exhibited necrotic activity. The highest necrotic index was observed for six strains (11.3 %) at 24 h and 8 isolates (15.1 %) at 48 h. Their indexes ranged from 39 to 41 % and from 45 to 69 %, respectively. Treatment of HEp-2 cells with cytochalasin D prior to infection, resulted in reduction of the apoptotic indexes to the range from 8.1 to 3.7 % for 19 strains (63 %) that exhibited invasion ability. Apoptosis of epithelial cells was also confirmed by the analysis of DNA fragmentation in cells infected with *S*. *haemolyticus* strains (Fig. 2). In the present study 2 (6.7 %) strains induced the intranucleosomal pattern with the size of about 180–200 bp in DNA extracted from cells at 24 h after infection. The number of strains that caused DNA fragmentation in HEp-2 cells increased to 17 (56.7 %) after 48 h of infection. Nuclear DNA fragmentation was observed when AI exceeded 51 %. The positive control of *Y. enterocolitica* O:8/1B induced DNA fragmentation in epithelial cells, whereas non-pathogenic *E. coli* K-12 C200, 28 (93.3 %) and 13 (43 %) of *S*. *haemolyticus* strains did not induce nuclear DNA fragmentation, respectively at 24 and 48 h post infection.

### Electron microscopic examination of infected epithelial cells

**Fig. 3:**
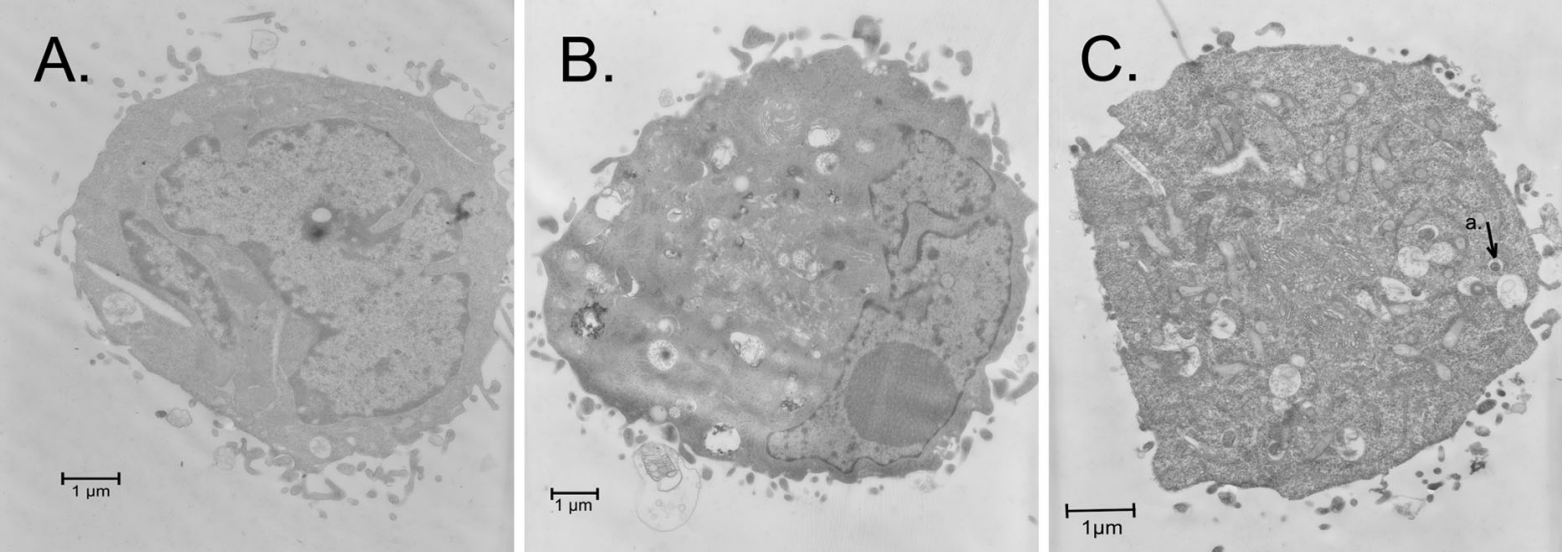
Transmission electron micrographs of epithelial cells. **a**—control, noninfected HEp-2 cell; **b**—chromatin condensation and nuclear condensation and fragmentation in cells infected with *S. haemolyticus* MPU Sh4 at 24 h; **c**—invaded bacteria into cellular cytoplasm were detected within endosomal vacuoles (**a**)

Transmission electron microscopy revealed that the negative control had an intact cell and organelle membrane and organelles and large nuclei (Fig. 3a). Infected with *S. haemolyticus* epithelial cells disclosed signs of apoptosis. The cells had an intact cell and nuclear membrane (Fig. 3b, c). The cells revealed chromatin condensation and decreasing of nuclear volume and its fragmentation (Fig. 3b), formation of apoptotic bodies inside the cells (Fig. 3b, c). The condensed chromatin in the apoptotic cells is localized to periphery (Fig. 3c) or one side of the nuclear fragments (Fig. 3b). Electron microscopy revealed that *S. haemolyticus* strains during adhesion, induced the formation of pseudopod structures of the epithelial cell wall, at the site of the bacterial attachment. The intracellular bacteria could be found residing in the membrane bound vesicles inside the cells. The vacuoles contained single bacteria (Fig. 3c).

### Activity of exoenzymes

Among thirty *S. haemolyticus* strains, eight (27 %) revealed lipolytic activity, 13 (43 %) produced lecithinase and 6 (20 %) were positive for proteinase activity.

## Discussion

The mechanisms of pathogenesis and virulence factors of *S*. *haemolyticus* strains have not yet been well defined. Therefore, this study was important for clarifying the pathogenicity of *S. haemolyticus* clinical isolates.

In this study, we observed a high rate of aminoglycoside resistance (63 %) of methicillin-resistant *S. haemolyticus* strains. The *aph*(3′)-*III* was found in 43 % and interpreted as the most frequently carried AME genes. Liakopoulos et al. (2011) suggested that the *aph(3′)*-*III* was also the most prevalent AME gene among *Staphylococcus aureus* and CNS isolated in Greece. In contrast, Ida et al. (2001) revealed that the *aph*(3′)-*III* genes were carried only by 8.9 % strains originated from Japan, whereas the most prevalent was the *ant*(4′)-*Ia* gene. In our studies, the *ant*(4′)-*Ia* gene was present only in six (20 %) strains. The *aac*(6′)/*aph*(2″) gene encoding bifunctional enzyme was present in 33 % of tested *S. haemolyticus* strains. In studies of staphylococci from 19 European hospitals, the *aac*(6′)/*aph*(2″)gene was present in 68 % isolates and was the most prevalent (Schmitz et al. 1999). The *aac*(6′)/*aph*(2″) genes have been found in *S. haemolyticus* isolated from human breast milk (Carneiro et al. 2004).

The epithelium is the first line of defence against infectious agents, playing a broad range of protective roles in the innate response to infection. Bacterial adherence to epithelial cells is a crucial step for pathogens to initiate and establish host colonization or to direct impact upon the eukaryotic cells (Hoffmann et al. 2011; Kim et al. 2010). Our results demonstrated that all isolates were able to adhere to HEp-2 cells. For 30 % isolates, the adhesion index was comparable to that of *Y. enterocolitica* O:8/1B, the enteroinvasive positive control. The highest adhesion ability was observed for 7 (67 %) strains isolated from blood and one isolate from skin and wound infections. Hyvönen et al. (2009) demonstrated that *S. haemolyticus* isolated had an adhesive ability to bovine mammary epithelial cells.

The ability of pathogenic bacteria to invade host cells and tissues is considered one of the most critical pathogenicity factors in persisting infections. Invasion of host cells allows pathogens to hide, persist within host tissues and to escape immune response (Hoffmann et al. 2011). This study revealed that *S. haemolyticus* strains were internalized by non-professional phagocytes. We observed that 19 of 30 strains (63 %) were invasive, with the invasion index higher than that of the nonpathogenic control. The highest invasion index, approximately from three to four times lower than that of *Y. enterocolitica* O:8/1B was observed for isolates originated from aspirate (MPU Sh2) and wounds (MPU Sh9, 4). Six isolates (20 %) revealed the lowest invasive ability, with efficiency higher than that of the *E. coli* nonpathogenic control. The results of *S. haemolyticus* invasion of epithelial cells were confirmed in transmission electron microscopy. We observed intracellular localization of the bacteria inside cytoplasmic vacuoles. Internalized bacteria can evade the host immune system and antibiotic treatment. The survival of *S. haemolyticus* strains in non-phagocytic cells, such as epithelial cells could have implication in bloodstream infections provoked by the isolates. It should be noted that 50 % of *S. haemolyticus* strains were isolated from blood samples (Krzymińska et al. 2012a). Szabados et al. (2008) suggested that *Staphylococcus saprophyticus* is internalized by the human urinary bladder carcinoma cell line. Foster et al. (2014) have identified a broad range of proteins that are covalently attached to peptidoglycan, which are known as cell wall-anchored proteins. These proteins revealed numerous functions, including adhesion to and invasion of host cells, biofilm formation and evasion of the host immune response. Hirschhausen et al. (2010) reported a novel mechanism of CNS internalization of the host cells, which is mediated by autolysins/adhesins from *Staphylococcus epidermidis* (AltE). The proteins have enzymatic and adhesive functions. In our results, the percentage of invading bacteria decreased after treatment of cytochalasin D prior to infection. The results suggested that invasion of *S. haemolyticus* strains to epithelial cells could be similar to phagocytosis that involved actin polymerization and subsequently the bacteria internalization into a vacuole. Sinha and Fraunholz (2010) reported that *S. aureus* revealed intracellular persistence in host cells. Whereas the bacteria were regarded as extracellularly located strains, some reports suggested their intracellular localization in host cells. The persistence of *S. aureus* in host cells is associated with their localization in vacuolar endosomal compartments. Internalization of *S. aureus* was inhibited by addition of cytochalasin D (Szabados et al. 2011).

We demonstrated that one of the virulence pathways of *S. haemolyticus* strains provoked contact-dependent cytotoxicity. In the study, all the strains displayed this activity, whereas little or no cytotoxicity was detected at the same time in culture supernatant of the strains. The results indicated that *S. haemolyticus* strains promote epithelial cell lysis by a virulence factor associated with a bacterial cell. We observed that 27 % of the strains revealed lipolytic activity, 43 % produced lecithinase and 20 % were positive for proteinase activity. We observed that strains that produced lipase, lecithinase and proteinase activities (MPU Sh4) or lipase and proteinase (MPU Sh9, 11, 13, 5, 2) exhibited the highest cell-contact cytotoxicity and invasion index. Some pathogenic strains may produce extracellular enzymes that could be responsible for host tissue degradation and development of an infection caused by the bacteria. Proteases can degrade host complement factors, mucins, and disrupt tight junctions between epithelial cells, leading to dissemination of the bacteria (Otto 2004). Proteases have been reported to be able to degrade fibrinogen, complement and other proteins, suggesting a role in the escape from innate defence system. Some CNS produced extracellular metalloprotease with elastase activity. The elastase that degrades human sIgA, IgM, serum albumin, fibronectin and fibrinogen is assumed to be a virulence factor (Otto 2004; Von Eiff et al. 2002). Lipases and phospholipases can target lipids in surfactants and host cell membranes. The enzymes may contribute to virulence by enabling the bacteria to persist in fatty secretions of human skin.

We observed that all S. *haemolyticus* strains exhibited cytotoxic activity of cell-free culture supernatant at 24 h after infection which suggested production of extracellular toxins. The highest activity in the range from 72.8 to 81.5 % was observed for 26.7 % isolates. Culture filtrates of the strains incubated at 56 °C for 20 min revealed a decrease in cytotoxic activity to epithelial cells that suggested that they produce heat-labile toxins. The results suggested that the strains displayed virulence factors involved in the infection process, showing the ability to damage host tissues as well as to evade the host defence system. Disruption of the epithelial barrier integrity may favour the bacterial tissue invasion and entry into the bloodstream. Previously, we noted that all S. *haemolyticus* strains were cytotoxic to macrophages (Krzymińska et al. 2012a). The important virulence factors that exert cytotoxic activity of CNS strains are extracellular enzymes and toxins: metalloprotease with elastase activity, cysteine and serine proteases, lipase, FAME and δ-toxins (Piette and Verschraegen 2009).

Our study revealed the fate of epithelial cells after bacterial adhesion and internalization. We demonstrated that one of the consequences of cell adhesion, invasion, and cytotoxic activity by *S.* *haemolyticus* strains is injury to the cells and cell death by apoptosis. We observed morphological changes of infected epithelial cells, including condensation of nucleus and nuclear chromatin, formation of apoptotic bodies. The highest apoptotic activity was revealed by strains isolated from wounds (MPU Sh4, 9) and urine (MPU Sh3). We observed that 56.7 % of strains caused fragmentation of nucleosomal DNA of infected cells to multimers which are a biochemical standard of apoptosis. The analysis of morphological changes and internucleosomal cleavage of the host cell DNA indicated that six strains isolated from blood, three from wounds and secretions, three from urine and medical devices and one strain originated from skin caused apoptosis of epithelial cells. All the strains which induced apoptosis of more than 58.4 % of epithelial cells were multiresistant to more than three different classes of antibiotics. Moreover, five strains revealed aminoglycoside resistance due to existence of genes *aac/aph* (MPU Sh2, 11, 25) and *aph*(3′)-*IIIa* (MPU SH10, 19). Seven strains did not harbor AME. The Pearson linear test revealed positive correlations between the AI of infected epithelial cells at 24 h and percentage of cell-cytotoxicity (r = 0.5, P < 0.01), extracellular cytotoxicity (r = 0.6, P < 0.01) and the invasion index (r = 0.2, P < 0.01). Current evidence suggested that apoptotic epithelial cells activate molecular signalling pathways for recognition by professional phagocytes and next removal of dead cells (Elliot and Ravichandran 2010).

The exact contribution of the *S. haemolyticus* virulence factors to pathogenesis is unclear. The present study showed that the importance of the strains as an emerging cause of nosocomial infections is connected with multiple strategies including antibiotics resistance, adhesion and invasion of human epithelial cells, production of extracellular enzymes and toxins that interact with the host cells. Moreover, the strains revealed evasion mechanisms and a survival strategy, residing in membrane-bound compartments and blocking essential non immune-mediated functions during infection.

## References

[CR1] Agerer F, Lux S, Michel A, Rohde M, Ohlsen K, Hauck CR (2005). Cellular invasion by *Staphylococcus aureus* reveals a functional link between focal adhesion kinase and cortactin in integrin-mediated internalization. J Cell Sci.

[CR2] Ardic N, Sareyyupoglu B, Ozyurt M, Haznedaroglu T, Ilga U (2006). Investigation of aminoglycoside modifying enzyme genes in methicillin-resistant staphylococci. Microbiol Res.

[CR3] Carneiro LAM, Queiroz MLP, Merquior VLC (2004). Antimicrobial-resistance and enterotoxin-encoding genes among staphylococci isolated from expressed human breast milk. J Med Microbiol.

[CR4] Choi CH, Lee EY, Lee YC, Park TI, Kim HJ, Hyun SH, Kim SA, Lee SK, Lee JC (2005). Outer membrane protein 38 of *Acinetobacter baumannii* localizes to the mitochondria and induces apoptosis of epithelial cells. Cell Microbiol.

[CR5] Elliot MR, Ravichandran KS (2010). Clearance of apoptotic cells: implication in health and disease. J Cell Biol.

[CR6] Foster TJ, Geoghegan JA, Ganesh VK, Höök M (2014). Adhesion, invasion and evasion: the many functions of the surface proteins of *Staphylococcus aureus*. Nat Rev Microbiol.

[CR7] Götz F, Dworkin M, Falkow S, Rosenberig E, Schleiferr K-H, Stackebrands E (2006). The genera *Staphylococcus* and *Micrococcus*. The prokaryotes.

[CR8] Hirschhausen N, Schlesier T, Schmidt MK, Götz F, Peters G, Heilmann C (2010). A novel staphylococcal internalization mechanism involves the major autolysin Atl and heat shock cognate protein Hsc 60 as host cell receptor. Cell Microbiol.

[CR9] Hoffmann C, Ohlsen K, Hauck CR (2011). Integrin-mediated uptake of fibronectin-binding bacteria. Eur J Cell Biol.

[CR10] Hyvönen P, Käyhkö S, Taponen S, von Wright A, Pyörälä S (2009). Effect of bovine lactoferrin on the internalization of coagulase-negative staphylococci into bovine mammary epithelial cells under in vitro conditions. J Dairy Res.

[CR11] Ida T, Okamoto R, Shimauchi C, Okubo T, Kuga A, Inoune M (2001). Identification of aminoglycoside-modifying enzymes by susceptibility testing: epidemiology of methicillin-resistant *Staphylococcus aureus* in Japan. J Clin Microbiol.

[CR12] Karasawa T, Wang X, Maegawa T, Michiwa Y, Kita H, Miwa K, Nakamura S (2003). C*lostridium sordellii* phospholipase C: gene cloning and comparison of enzymatic and biological activities with those of *Clostridium perfringens* and *Clostriduim bifermentans* phospholipase C. Infect Immun.

[CR13] Kim M, Ashida H, Ogawa M, Yoshikawa Y, Mimuro H, Sasakawa C (2010). Bacterial interactions with the host epithelium. Cell Host Microbe.

[CR14] Krzymińska S, Koczura R, Mokracka J, Puton T, Kaznowski A (2010). Isolates of the *Enterobacter**cloacae* complex induce apoptosis of human intestinal epithelial cells. Microb Pathog.

[CR15] Krzymińska S, Szczuka E, Kaznowski A (2012). *Staphylococcus haemolyticus* strains target mitochondria and induce caspase-dependent apoptosis of macrophages. Antonie Van Leeuwenhoek.

[CR16] Krzymińska S, Ochocka K, Kaznowski A (2012). Apoptosis of epithelial cells and macrophages due to nonpigmented *Serratia marcescens* strains. Sci World J.

[CR17] Kumar D, Kumar L, Nagar S, Raina C, Parshad R, Gupta VK (2012). Screening, isolation and production *Bacillus* sp. strain DVL2 and its potential evaluation in esterification and resolution reactions. Arch Appl Sci Res.

[CR18] Liakopoulos A, Foka A, Vourli S, Zerva L, Tsiapara F, Protonotariou E (2011). Aminoglycoside-resistant staphylococci in Greece: prevalence and resistance mechanisms. Eur J Clin Microbiol Infect Dis.

[CR19] Lucas AL, Manna AC (2013). Phenotypic characterization of sarR mutant in *Staphylococcus aureus*. Microb Pathogen.

[CR20] Otto M (2004). Virulence factors of coagulase-negative staphylococci. Front Biosci.

[CR21] Piette A, Verschraegen G (2009). Role of coagulase-negative staphylococci in human disease. Vet Microbiol.

[CR22] Rocha-de-Souza CM, Berent-Maoz B, Mankuta D, Moses AE, Levi-Schafer F (2008). Human mast cell activation by *Staphylococcus aureus*: interleukin-8 and tumor necrosis factor alpha release and the role of toll-like receptor 2 and CD48 molecules. Infect Immun.

[CR23] Schmitz F, Fluit AC, Gondolf M, Beyrau R, Lindenlauf E, Verhoef J, Heinz HP, Jones ME (1999). The prevalence of aminoglycoside resistance and corresponding genes in clinical isolates of staphylococci from 19 European hospitals. J Antimicrob Chemother.

[CR24] Sinha B, Frauholz M (2010). *Staphylococcus aureus* host cell invasion and post-invasion events. Int J Med Microbiol.

[CR25] Szabados F, Kleine B, Anders A, Kaase M, Sakinç T, Schmitz I, Gatermann S (2008). *Staphylococcus saprophyticus* ATCC 15305 is internalized into human urinary bladder carcinoma cell line 5637. FEMS Microbiol Lett.

[CR26] Szabados F, MarlinghansL KM, Neumann S, Kaase M, Gatermann S (2011). Fbl is not involved in the invasion of eukaryotic epithelial and endothelial cells by *Staphylococcus lugdunensis*. FEMS Microbiol Lett.

[CR27] Takueschi F, Watanabe S, Baba T (2005). Whole genome sequencing of *Staphylococcus**haemolyticus* uncovers extreme plasticity of its genome and the evolution of human-colonizing staphylococcal species. J Bacteriol.

[CR28] Vakulenko SB, Mobashery S (2003). Versatility of aminoglycosides and prospects for their future. Clin Microbiol Rev.

[CR29] Von Eiff C, Peters G, Heilmann C (2002). Pathogenesis of infections due to coagulase negative staphylococci. Lancet Infect Dis.

[CR30] You I, Kariyama R, Zervos MJ, Kumon H, Chow JW (2000). In-vitro activity of arbekacin alone and in combination with vancomycin against gentamicin- and methicillin-resistant *Staphylococcus aureus*. Diagn Microbiol Infect Dis.

